# Identification of a nomogram based on long non-coding RNA to improve prognosis prediction of esophageal squamous cell carcinoma

**DOI:** 10.18632/aging.102697

**Published:** 2020-01-24

**Authors:** Wenli Li, Jun Liu, Hetong Zhao

**Affiliations:** 1 Reproductive Medicine Center, Yue Bei People’s Hospital, Shantou University Medical College, Shaoguan, Guangdong, China; 2 Department of Clinical Laboratory, Yue Bei People’s Hospital, Shantou University Medical College, Shaoguan, Guangdong, China; 3 Department of Traditional Chinese Medicine, Changhai Hospital, Naval Military Medical University, Shanghai, China

**Keywords:** esophageal squamous cell carcinoma, long non-coding RNA, signature, nomogram, weighted gene co-expression network analysis

## Abstract

Purpose: Esophageal squamous cell carcinoma (ESCC) remains a common aggressive malignancy in the world. Several long non-coding RNAs (lncRNAs) are reported to predict the prognosis of ESCC. Therefore, an in-depth research is urgently needed to further investigate the prognostic value of lncRNAs in ESCC.

Results: From the training set, we identified a eight-lncRNA signature (including AP000487, AC011997, LINC01592, LINC01497, LINC01711, FENDRR, AC087045, AC137770) which separated the patients into two groups with significantly different overall survival (hazard ratio, HR = 3.79, 95% confidence interval, 95% CI [2.56-5.62]; *P* < 0.001). The signature was applied to the validation set (HR = 2.73, 95%CI [1.65-4.53]; *P* < 0.001) and showed similar prognostic values. Stratified, univariate and multivariate Cox regression analysis indicated that the signature was an independent prognostic factor for patients with ESCC. A nomogram based on the lncRNAs signature, age, grade and stage was developed and showed good accuracy for predicting 1-, 3- and 5-year survival probability of ESCC patients. We found a strong correlation between the gene significance for the survival time and T stage. Eight modules were constructed, among which the key module most closely associated with clinical information was identified.

Conclusions: Our eight-lincRNA signature and nomogram could be practical and reliable prognostic tools for esophageal squamous cell carcinoma.

Methods: We downloaded the lncRNA expression profiles of ESCC patients from Gene Expression Omnibus (GEO) and The Cancer Genome Atlas (TCGA) datasets and separated to training and validation cohort. The univariate, least absolute shrinkage and selection operator (LASSO) and multivariate Cox regression analysis were used to identify a lncRNA-based signature. The predictive value of the signature was assessed using the Kaplan-Meier method, receiver operating characteristic (ROC) curves and area under curve (AUC). Weighted gene co-expression network analysis (WGCNA) was applied to predict the intrinsic relationship between gene expressions. In addition, we further explored the combination of clinical information and module construction.

## INTRODUCTION

Esophageal cancer (EC) is a leading malignancy worldwide, with approximately 572,000 new patients and 508,000 deaths annually [1]. Nearly 90% of patients with EC in Eastern Asia have esophageal squamous cell carcinoma (ESCC) [2–4]. Due to its rapid progress and insensitive to chemotherapy, the outcome of ESCC remains extremely poor. In spite of the development of surgical and medical management, many ESCC patients suffered from diagnosed at late stage. The pathogenesis of ESCC is a multi-step process, including several stages until ultimately carcinoma [5]. Therefore, focusing on the molecular mechanisms underlying the initiation and development of ESCC will help reveal promising diagnostic biomarkers and novel therapeutic targets.

The development of high-throughput sequencing technologies has improved our understanding of the heterogeneity and molecular basis underlying the ESCC [6]. LncRNAs, defined as non-protein-coding RNA transcripts (>200 nt), are reported to play a vital role recently in the genomics era [7]. To date, accumulating evidences supported the potential biomarkers of lncRNAs in a large range of cancers including ESCC. A few lncRNAs have been suggested as oncogenic roles in ESCC so far. For instance, increased expression of FMR1-AS1 has recently been reported to be a poor marker in female esophageal carcinoma [8], while LINC01503 is confirmed to be significantly higher level in ESCC tumors and correlated to poorer survival times of patients [9]. With the increasing progress in bioinformatics, a wide spectrum of disease prediction and investigation of molecular mechanisms have come to light. Therefore, the biological significance of lncRNAs in the development of ESCC needs further research.

In our research, we intended to develop a concise lncRNA-based signature and nomogram to improve prognostic value of ESCC via integrated bioinformatics approaches.

## RESULTS

### DELs identification

**Figure 1:**
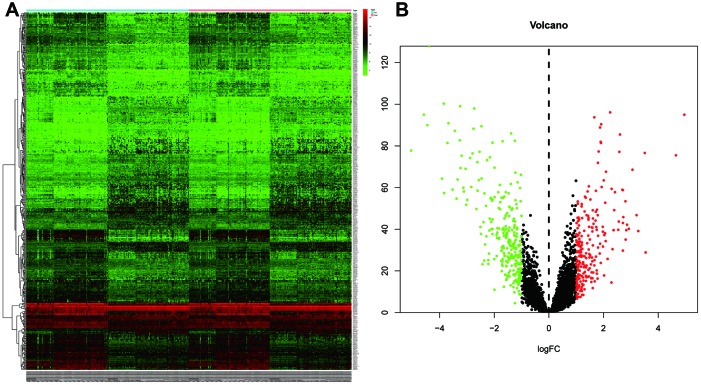
**Identification of differentially expressed lincRNAs (DELs) by using “edgeR” and “DEseq” R package.** (**A**) The heatmap of the DELs in ESCC when compared with normal tissue. (**B**) Volcano plot shown the expression change in ESCC when compared with normal tissue. An absolute log2 fold change (FC) > 1 and an adjusted P value of < 0.05 cutoff was used to defined DELs.

All 502 lincRNAs was identified as differentially expressed lincRNAs (DELs) between tumor tissues and adjacent normal tissues, including 223 up-regulated and 279 down-regulated genes (Figure 1A and 1B).

### Construction and validation of the eight-lincRNA signature

**Figure 2:**
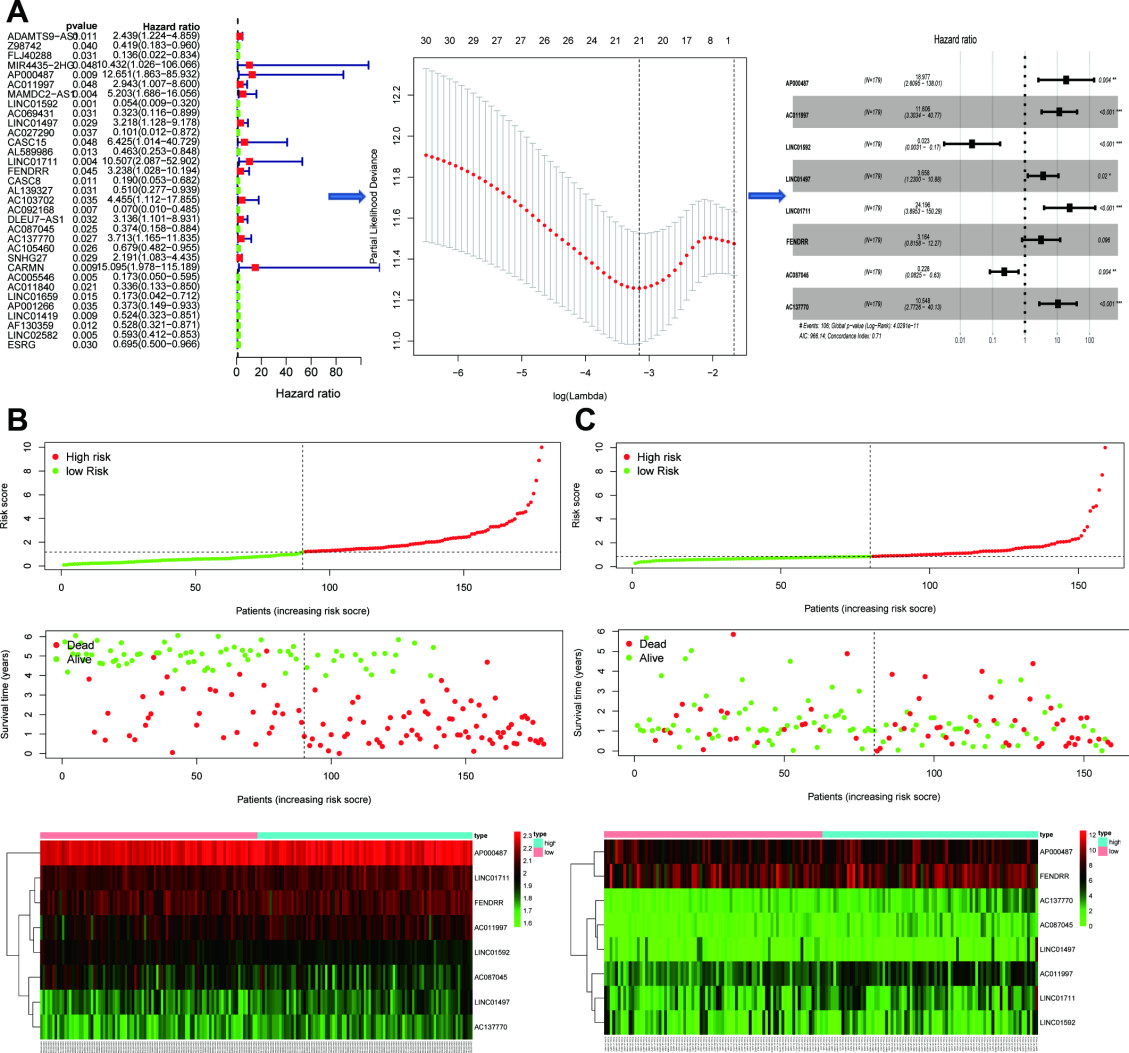
**Establishment and validation of the eight-lincRNA prognostic signature.** (**A**) The procedure of the establishment of the prognostic signature. (**B**–**C**) Correlation between the prognostic signature and the overall survival of patients in the GEO cohort (**B**) and TCGA (**C**) cohorts. The distribution of risk scores (upper), survival time (middle) and lincRNA expression levels (below). The black dotted lines represent the median risk score cut-off dividing patients into low- and high-risk groups. The red dots and lines represent the patients in high-risk groups. The green dots and lines represent the patients in low-risk groups.

**Figure 3:**
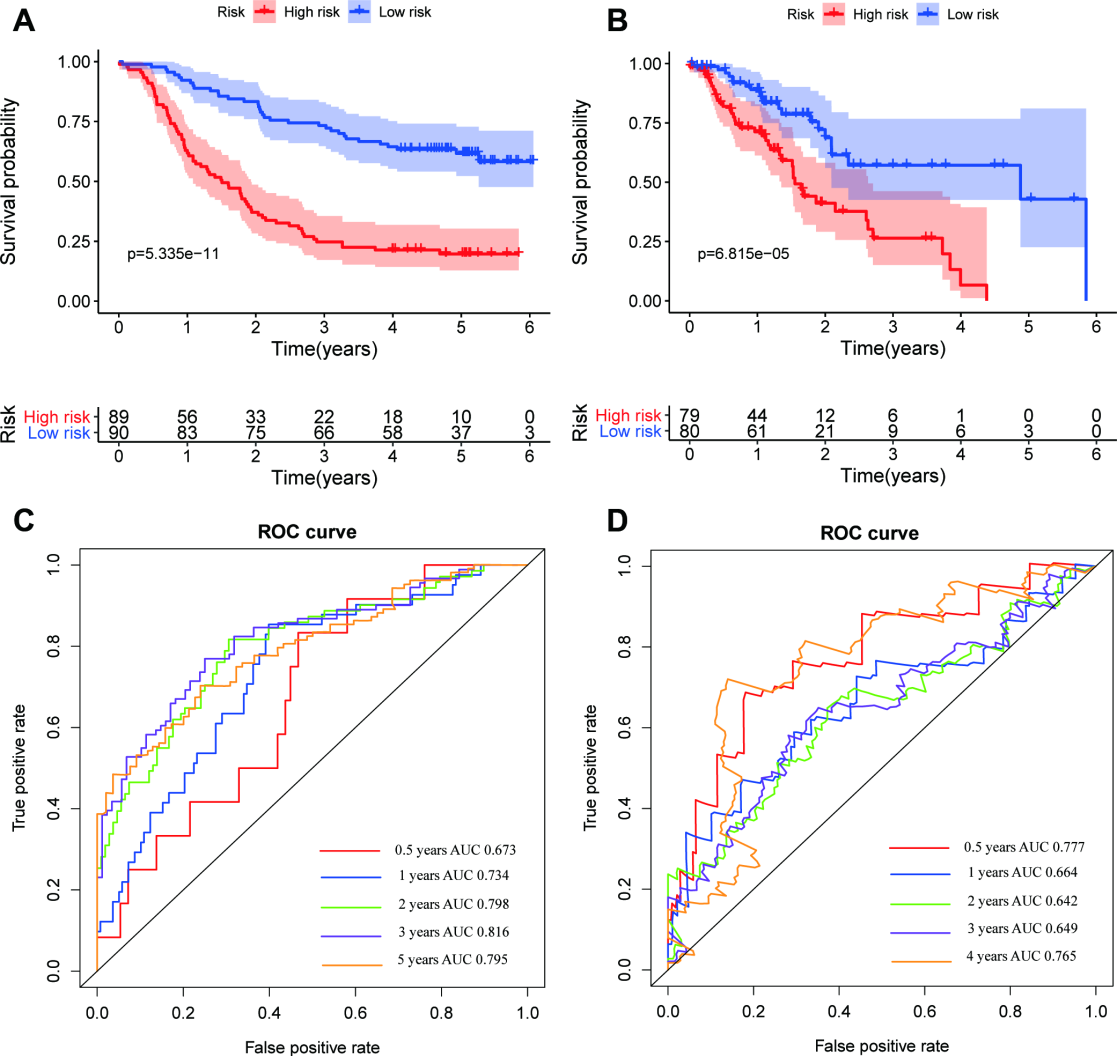
**Kaplan–Meier survival and ROC curves of the eight-lincRNA prognostic signature.** (**A**, **B**) Kaplan-Meier survival curves of overall survival among risk stratification groups in the GEO (**A**) and TCGA (**B**) set. (**C**, **D**) ROC curves with calculated AUCs for risk prediction in 0.5-, 1-, 2-, 3-, 5-years in the GEO (**C**) and TCGA (**D**) sets.

The DELs of the training set (GSE53625) were exposed to univariate COX regression and 33 DELs related to the OS (*P* < 0.05) were measured as predictive lincRNAs for LASSO analysis (Table 1). Finally, eight lincRNAs were selected to construct a risk signature for ESCC using multivariate COX regression. The risk score of the signature for OS was identified: risk score = 18.977 × (expression level of AP000487) + 11.606 × (expression level of AC011997) + 0.023 × (expression level of LINC01592) + 3.658 × (expression level of LINC01497) + 24.196 × (expression level of LINC01711) + 3.164 × (expression level of FENDRR) + 0.228 × (expression level of AC087045) + 10.548 × (expression level of AC137770). As above, it was suggested that the lncRNAs in the signature were all risk factors for OS. Their coefficients indicated their impact on OS prediction. For example, the influence of LINC01711 was greatest while that of LINC01592 was least. All patients were separated to high- and low-risk sets based on median risk score in the GEO (Figure 2B) and TCGA (Figure 2C) cohorts. The patients’ status, survival time, and lincRNA expression levels are shown in Figure 2B (GEO) and 2C (TCGA). The survival analysis presented that the OS of low-risk set was better than that of high-risk set in the GEO cohort (hazard ratio, HR = 3.79, 95% confidence interval, 95% CI [2.56-5.62]; *P* < 0.001) (Figure 3A). The results were consistent in the TCGA cohort (HR = 2.73, 95%CI [1.65-4.53]; *P* < 0.001) (Figure 3B). The area under the ROC curve (AUC) for 0.5-, 1-, 3-, and 5-year OS were 0.673, 0.734, 0.798, 0.816, 0.795 and 0.777, 0.644, 0.642, 0.649, 0.765 in the TCGA and GEO cohorts, respectively. Together, it was indicated that the signature showed an excellent performance for OS prediction.

**Table 1:** Independent prognostic genes in the signature.

**id**	**coef**	**HR**	**HR.95L**	**HR.95H**	**pvalue**
AP000487	2.943225	18.97694	2.609465	138.0069	0.003644
AC011997	2.451485	11.60557	3.303366	40.77331	0.000131
LINC01592	-3.7645	0.023179	0.003134	0.171457	0.000227
LINC01497	1.29683	3.657683	1.229963	10.87728	0.01969
LINC01711	3.186168	24.19554	3.895292	150.2902	0.000628
FENDRR	1.151995	3.164499	0.815825	12.27475	0.095784
AC087045	-1.47668	0.228395	0.082496	0.632323	0.004481
AC137770	2.355955	10.54819	2.77264	40.1294	0.000549

Abbreviation: HR, hazard ratio; CI, confidential interval.

### Subgroup, univariate and multivariate COX regression analysis of the signature

**Figure 4:**
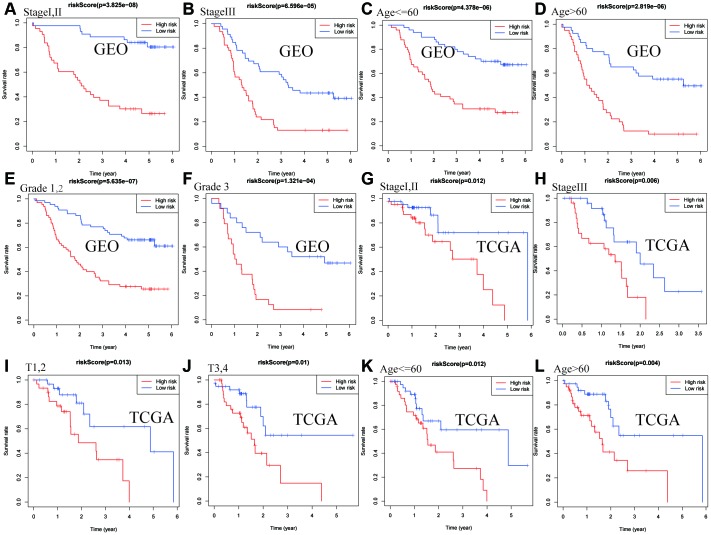
**Confirmation of the signature stratified by different clinical factors in the GEO and TCGA cohorts.** Kaplan–Meier survival for OS in subgroups stratified by stage I,II (**A**) stage III (**B**) age≤60 (**C**) age > 60 (**D**) grade 1,2 (**E**) grade 3 (**F**) in the GEO cohort, and stage I,II (**G**) stage III (**H**) T1,2(**I**) T3,4(**J**) age≤60 (**K**) age > 60 (**L**) in the TCGA cohort.

As shown in Figure 4, the risk score identified by prognostic signature assists as a helpful biomarker for OS prediction in different subgroups, including stage I,II (*P* < 0.0001), stage III (*P* < 0.0001), age≤60 (*P* < 0.0001), age > 60 (*P* < 0.0001), grade1,2 (*P* < 0.0001), grade 3 (P < 0.0001) in the GEO cohort, and stage I,II (*P* = 0.012), stage III (*P* = 0.006), T1,2 (*P* = 0.013), T3,4 (*P* = 0.01), age ≤ 60 (*P* = 0.012), age > 60 (*P* = 0.004) in the TCGA cohort, respectively.

**Figure 5:**
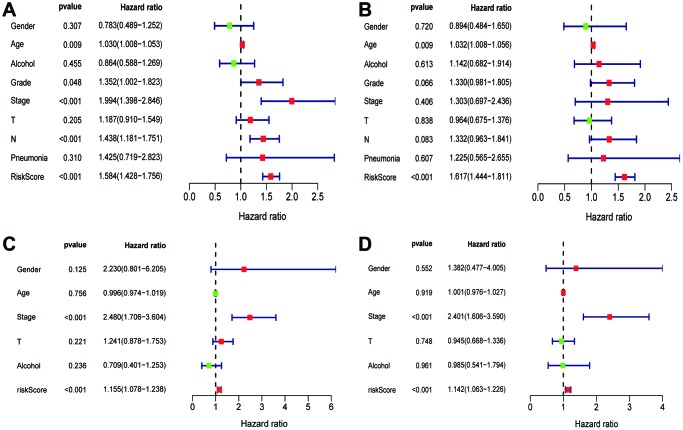
**Univariate and multivariate Cox regression analyses of clinical factors associated with overall survival.** (**A**-**B**) Univariate Cox regression analyses of clinical factors associated with overall survival in the TCGA (**A**) and GEO (**C**) set. (**C**-**D**) Multivariate Cox regression analyses of clinical factors associated with overall survival in the TCGA (**B**) and GEO (**D**) sets.

The univariate Cox regression showed that grade, stage, N stage and risk score in the TCGA cohort (grade: *P* =0.048, stage: *P* < 0.001; N stage: *P* < 0.041, risk score: *P* < 0.001; Figure 5A), and stage, risk score in the GEO cohort (stage: *P* < 0.001; risk score: *P* < 0.001; Figure 5C) were predictors for OS. Moreover, multivariate Cox regression analysis confirmed that age (HR = 1.032, 95% CI [1.008–1.056]; *P* = 0.009; Figure 5B) and risk score (HR = 1.617; 95% CI [1.444–1.811]; *P* < 0.001; Figure 5B) were significant independent risk factors in the TCGA cohort. Multivariate Cox regression further showed that stage (HR = 2.401, 95% CI [1.606–3.590]; *P* < 0.001; Figure 5D) and risk score (HR = 1.142; 95% CI [1.063–1.226]; *P* < 0.001; Figure 5D) was significant independent risk factors in the GEO cohort. These data indicated that the signature was an independent risk factor of ESCC.

### Nomogram construction

**Figure 6:**
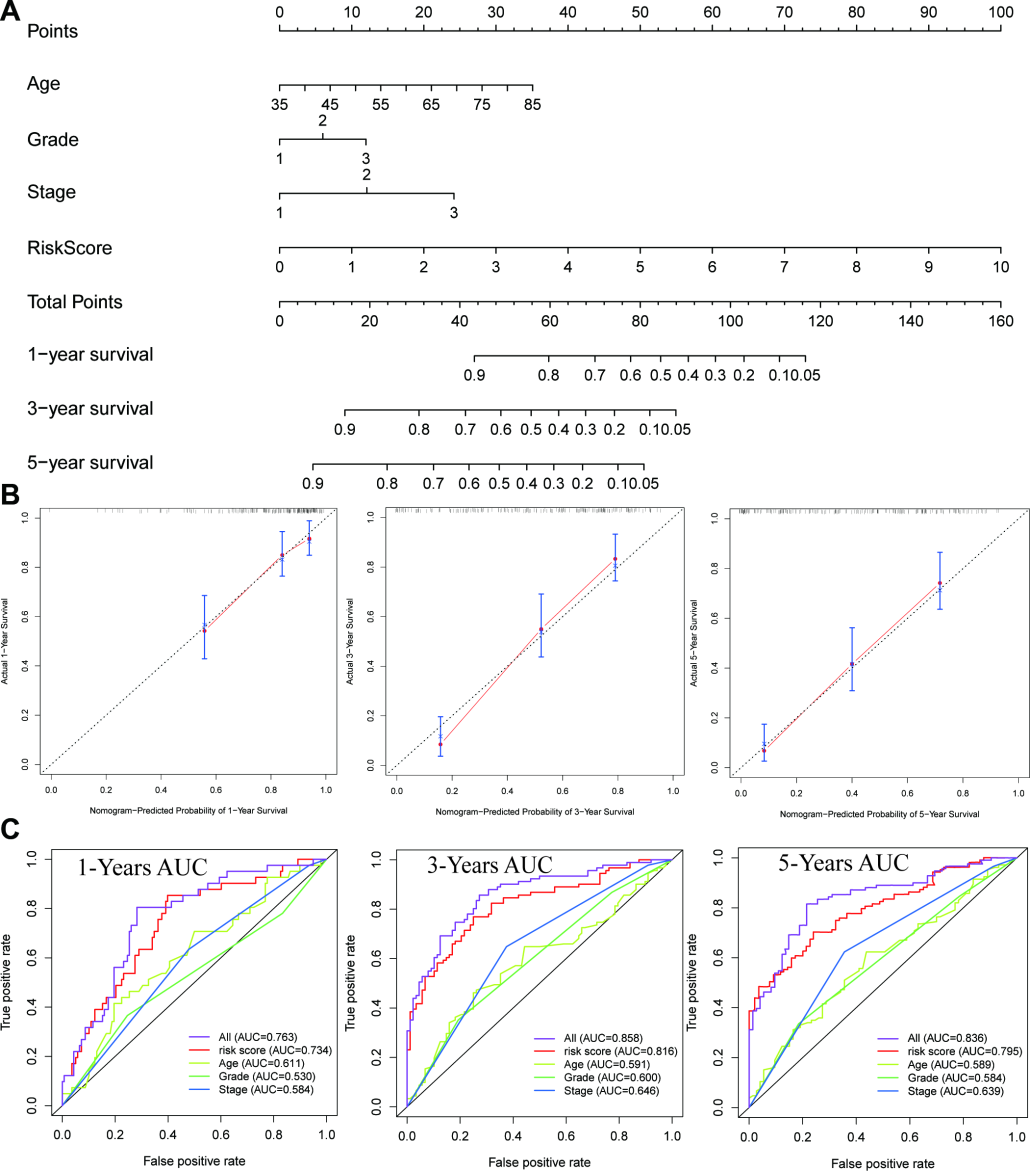
**Construction of a nomogram for overall survival prediction in ESCC.** (**A**) The nomogram consists of age, grade, stage and the risk score based on the eight-lncRNA signature. (**B**) Calibration curves of the nomogram for the estimation of survival rates at 1-, 3-, 5- year. (**C**) The Kaplan-Meier curves of the risk subgroups stratified by the tertiles of total points derived from the nomogram.

Based on the prognostic signature and clinical factors, such as age, grade and stage, a nomogram was constructed (Figure 6A). The calibration curve was used to describe the prediction value of the nomogram and the 45-degree line indicated the actual survival outcomes. The results for predicting 1-, 3- and 5-year OS showed that the nomogram-predicted survival closely matched with the best prediction performance (Figure 6B). The 1-year AUC was 0.734 for nomogram, and 0.611 for age, 0.530 for grade, 0.584 for stage. The 3-year AUC was 0.816 for nomogram, and 0.591 for age, 0.600 for grade, 0.646 for stage. Moreover, the 5-year AUC was 0.795 for nomogram, and 0.589 for age, 0.584 for grade, 0.639 for stage. These findings showed that compared with a single clinical factor, the nomogram combined the signature and clinical factors had great predictive accuracy.

### WGCNA

**Figure 7:**
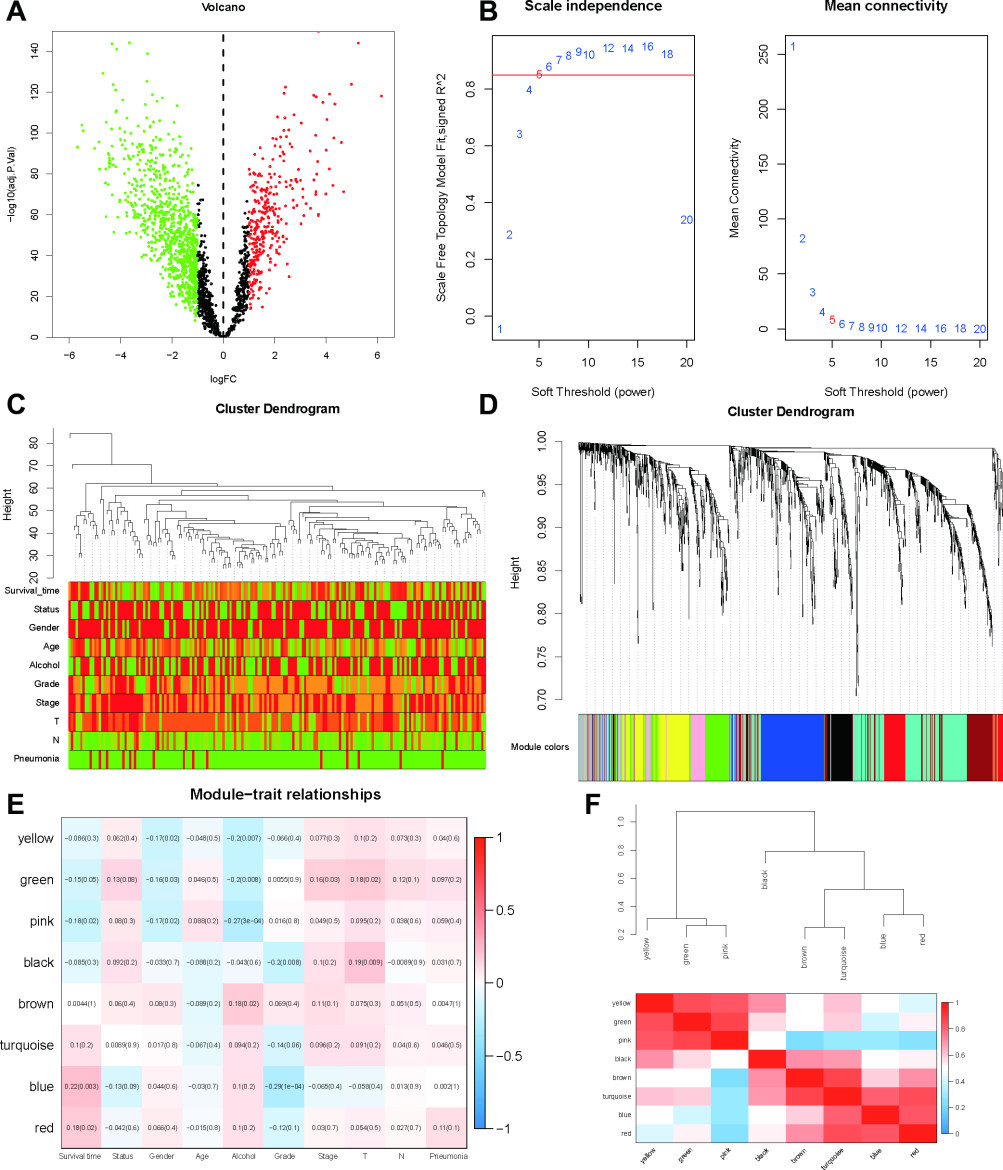
**WGCNA analysis.** (**A**) Volcano plot of differentially expressed genes associated with lincRNAs in the signature (correlation coefficient < 0.7, P < 0.001). (**B**) Analysis of the scale-free topology model fit index for various soft-thresholding powers (β) and the mean connectivity for various soft-thresholding powers. In all, 5 was the most fit power value. (**C**) Dendrogram of the genes and different clinical factors of ESCC (survival time, status, gender, age, alcohol, grade, stage, T, N, Pneumonia). (**D**) Dendrogram of the gene modules based on a dissimilarity measure. The branches of the cluster dendrogram correspond to the different gene modules. Each piece of the leaves on the cluster dendrogram corresponds to a gene. (**E**) Module-trait relationships. Heatmap of the correlation between module eigengenes and clinical characteristics of ESCC. (**F**) Hierarchical clustering and heatmap of the hub gene network.

**Figure 8:**
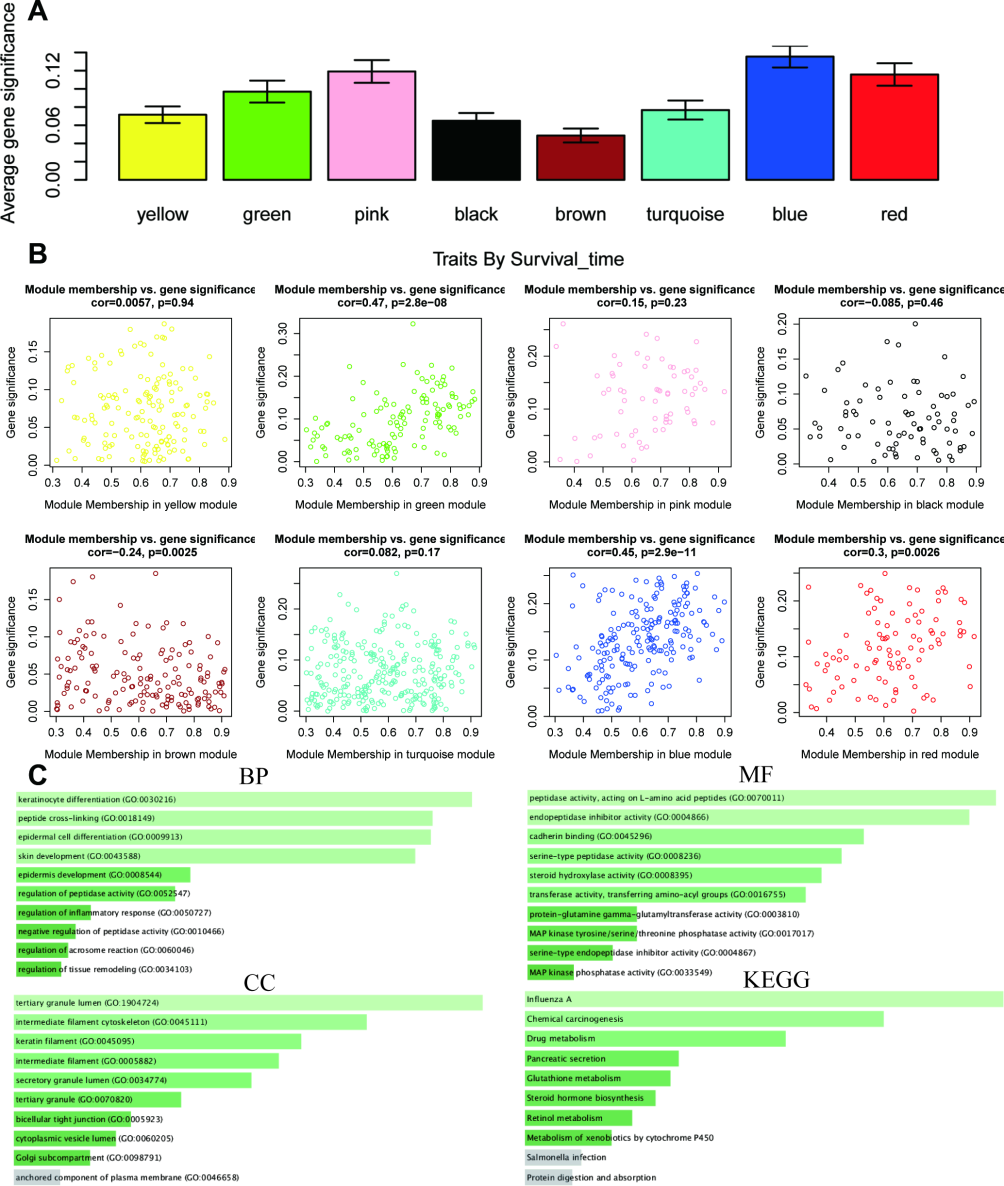
**The correlation between the genes in the modules and survival time.** (**A**) Distribution of mean gene significance and standard deviation with survival time in the modules of ESCC. (**B**) Scatter plot of module eigengenes in eight modules. (**C**) GO and KEGG pathway enrichment of eight modules. GO enrichment contains three categories including biological process, cellular component and molecular function.

WGCNA was used to develop a gene co-expression system to select biologically meaningful gene modules which are related to the lincRNAs in the signature. We set the cut-off as Person correlation coefficient > 0.6 and *P* < 0.001 to screen genes co-expression with lincRNAs. Then, differential expressed gene (DEG) analysis was performed among these genes. Figure 7A shows the volcano plot of DEGs. To create a scale-free system, the scale-free topology fit index reached 0.8 by setting the soft threshold power value beta to 5 (Figure 7B). In addition, genes with similar patterns was located in different modules by average linkage clustering (Figure 7C). The minimum cluster size was identified as 30 per module. The dynamic shear method was performed to determine the gene module. We calculated module eigengene (ME) which was the overall gene expression level of corresponding modules and clustered them based on their correlation in order to explore co-expression similarity of all modules (Figure 7D). To analyze the connection of gene modules and clinical characteristics, eigengenes were calculated correlations with clinical factors, such as survival time, status, gender, age, alcohol, grade, stage, T, N, pneumonia. Finally, the robust correlation was identified between the gene significance and the survival time, T stage (Figure 7E). The eight modules were generally separated into two clusters (Figure 7F). Gene significance were also determined to evaluate the correlation between gene expression and survival time (Figure 8A). To further analyze genes in these modules, we found a strong correlation between the gene significance for the survival time and Module Membership in the green module (cor = 0.47, *P* = 2.8e − 08), the brown module was negatively correlated with the survival time (cor = −0.24, *P* = 0.0025), the blue module was positively correlated with the survival time (cor = 0.45, *P* = 2.9e − 11), and the red module was positively correlated with the survival time (cor = 0.3, *P* = 0.0026) (Figure 8B). The function enrichment analysis was performed to explore the GO database and KEGG pathway in which are involved (Figure 8C). The results indicated that the biological process mainly involved in keratinocyte differentiation, peptide cross-linking, epidermal cell differentiation, and skin development and so on. The results showed that the molecular function was related to peptidase activity, acting on L-amino acid peptides, endopeptidase inhibitor activity, cadherin binding and serine-type peptidase activity. The cell components which was correlated to the results included tertiary granule lumen, intermediate filament cytoskeleton, keratin filament, and intermediate filament. Furthermore, KEGG pathway functional enrichment showed that influenza A, chemical carcinogenesis and drug metabolism were mainly related to the genes in these modules.

**Figure 9:**
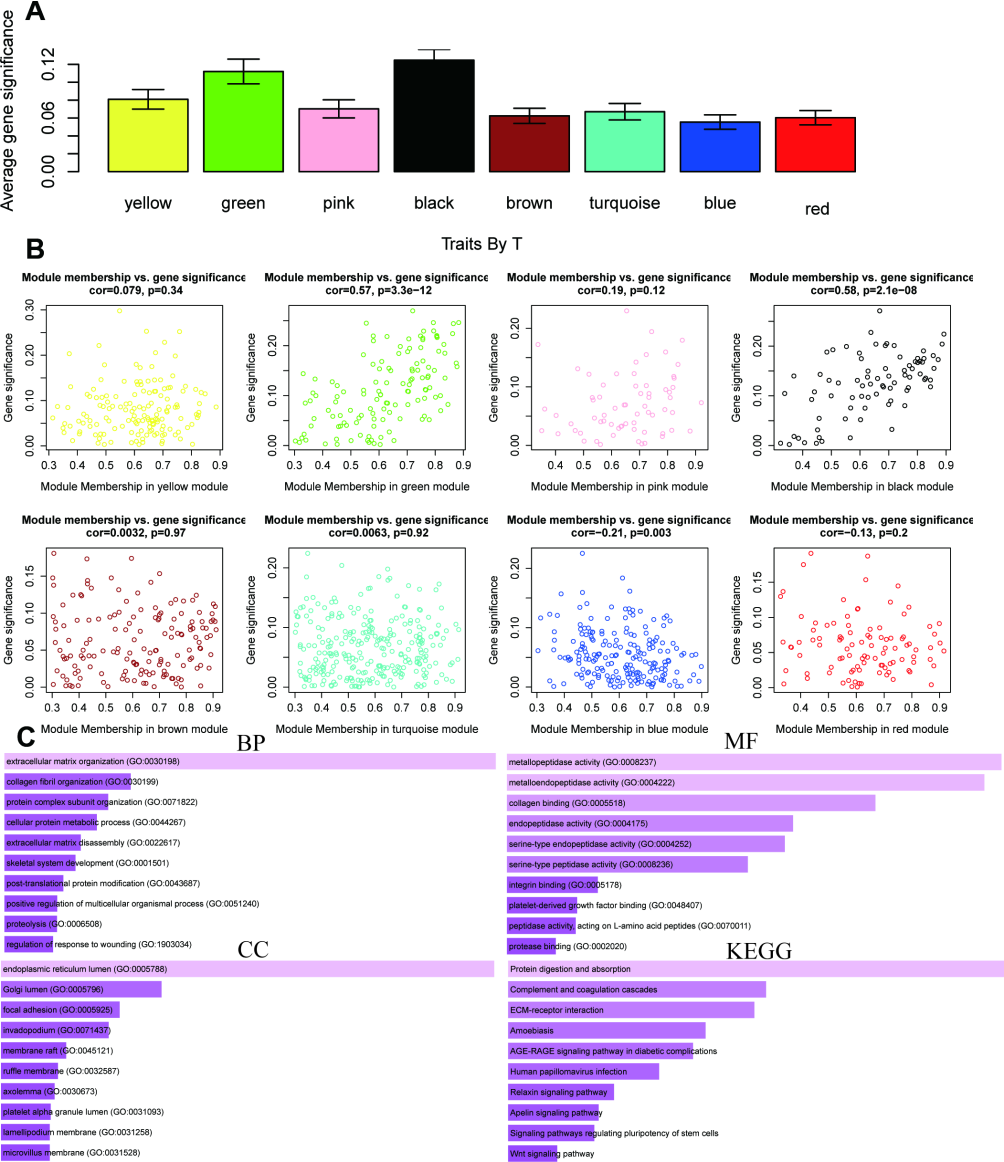
**The correlation between the genes in the modules and T stage.** (**A**) Distribution of mean gene significance and standard deviation with survival time in the modules of ESCC. (**B**) Scatter plot of module eigengenes in eight modules. (**C**) GO and KEGG pathway enrichment of eight modules. GO enrichment contains three categories including biological process, cellular component and molecular function.

The same analysis was completed to assess the correlation between gene expression and T stage (Figure 9A). Thus, we found a strong correlation between the gene significance for the T stage and Module Membership in the green module (cor = 0.57, *P* = 3.3e − 12), the black module was positively correlated with the T stage (cor = − 0.24, *P* = 2.1e – 8), and the blue module was negatively correlated with the T stage (cor = 0.21, *P* = 0.003) (Figure 9B). The function enrichment analysis was also performed to explore the GO database and KEGG pathway in which are involved (Figure 9C). The results indicated that the biological process mainly involved in extracellular matrix organization. The results showed that the molecular function was related to metallopeptidase activity, metalloendopeptidase activity and collagen biding. The cell components which was mainly correlated to the results included endoplasmic reticulum lumen. Moreover, KEGG pathway functional enrichment showed that protein digestion and absorption was mainly related to the genes in these modules.

## DISCUSSION

ESCC remains a serious burden on health system worldwide. Traditional algorisms such as tumor-node-metastasis (TNM) staging system failed to consider the genetic alterations of ESCC. Thus, in view of the high heterogeneity of ESCC, investigation of novel biomarkers and models is necessary. Recently, lincRNA-based signatures have received much focus and revealed excellent potential in prognosis prediction of numerous cancers [10, 11].

In the present study, we identified DELs between tumor and normal tissues in GEO data, and confirmed lincRNAs significantly correlated with prognosis using univariate COX regression and LASSO analysis. Finally, eight lincRNAs (AP000487, AC011997, LINC01592, LINC01497, LINC01711, FENDRR, AC087045, AC137770) were selected to build a prognostic signature for ESCC. A robust nomogram consisted of the 8-lncRNAs signature, age, grade and stage was constructed for prognostic prediction of ESCC patients. Furthermore, the AUC value of the signature-based nomogram was better than the AUC values of age, grade and stage in 1-, 3-, 5- years.

In this study, we identified 8 key prognostic lncRNAs of ESRCC patients, however, no review had been studied about intriguing mechanisms of these lncRNAs except FENDRR. FENDRR is transcribed from the FOXF1 promoter and considered to be one of the favorable lncRNA biomarkers for various cancers, such as liver cancer [12], lung squamous cell carcinoma [13], bladder cancer [14], gastric cancer [15] and lung adenocarcinoma [16]. lncRNA FENDRR was first identified associated with chromatin-modifying complexes in 2009 [17]. FENDRR could suppress the progression of NSCLC by regulating miR-761 and TIMP2 [18]. Consistent with the previously published papers, the expression levels of FENDRR were found positively related to the risk score of ESCC patients in our research. It is worth noting that patients with high FENDRR expression appeared to be closed to poor prognosis in ESCC.

lncRNA-based signature is a novel tool which could provide simple and accurate clinical outcome prediction. So far, three lncRNA-based signatures have been developed for ESCC through bioinformatics methods [19–21]. For instance, a three-lncRNA signature was established based on multivariable Cox regression analysis and could precisely predict OS and disease-free survival (DFS) for ESCC [19]. Another nine-lncRNA signature was constructed by random forest algorithm and support vector machine algorithm and identified to predict the tumor stage and patient survival rate [21]. The third seven-lncRNA signature was identified by random survival forest algorithm and Cox regression analysis and this signature combined with TNM showed better prognostic predict capability than either alone [20]. However, these signatures were failing to describe the clinical significance of the signature. As far as we know, clinical factors such as gender and grade could also have an influence on the OS. These factors need to be included to improve the prediction accurate.

Nomogram is a commonly used tool in oncology which can create an individual probability by integrating diverse prognostic and determinant variables according to corresponding clinical characteristics [22]. Several nomograms were constructed to guide individualized treatment based on clinicopathological risk factors in renal cell carcinoma [23], breast cancer [24] and colorectal cancer [25]. In this study, a prognostic nomogram combined signature with clinical factors was settled. The clinical factors in the nomogram are not affected by researchers and can be easily obtained. What’s more, our nomogram had a better predictive accuracy than that of each factor alone.

WGCNA is a useful tool to investigate the molecular mechanisms of many malignancies, such as breast cancer [26] and colon cancer [27]. In present research, WGCNA was performed to analyze the genes associated with the lincRNAs in the signature. We used this method to transform the expression profiles from these genes to 8 modules. In these modules, we further focused on gene pivots highly related to various clinical features. Functional enrichment of key module genes was also analyzed.

In summary, our eight-lincRNA signature and nomogram could be practical and reliable prognostic tools for esophageal squamous cell carcinoma. They can offer incremental clinical value over traditional staging system for overall survival prediction of ESCC, which can utilize treatment decisions.

## MATERIALS AND METHODS

### Data collection

The RNA-sequencing and clinical information for ESCC were acquired from the Gene Expression Omnibus (GEO) database (https://www.ncbi.nlm.nih.gov/geo/) as the training cohort, and The Cancer Genome Atlas (TCGA) database (http://cancergenome.nih.gov/) as the validation cohort. GSE53625 from GEO was conducted by GPL18109 (Agilent-038314 CBC Homo sapiens lncRNA + mRNA microarray V2.0) [28]. The clinical features of patients with ESCC in the TCGA and GEO cohorts are presented in Table 2.

**Table 2:** The clinical features of patients with ESCC in the TCGA and GEO cohorts.

**GEO**					
Gender	Female	33	Stage	I	10
	Male	146		II	77
Age	<=60	99		III	92
	>60	80	T	T1	12
Alcohol	No	73		T2	27
	Yes	106		T3	110
Grade	G1	32		T4	30
	G2	98	N	N0	83
	G3	49		N1	62
Pneumonia	No	164		N2	22
	Yes	15		N3	12
**TCGA**					
Gender	Female	23	T	T1	28
	Male	136		T2	37
Age	<=60	81		T3	75
	>60	78		T4	4
Grade	G1	16		unknow	15
	G2	65	Stage	I	16
	G3	43		II	68
	GX	35		III	48
Alcohol	No	46		IV	8
	Yes	110		unknow	19

### Identification of differentially expressed lincRNAs in ESCC

The RNA-sequencing data were normalized with Expectation-Maximization algorithm (log2 transformation). Then 37501 lincRNAs were annotated. The differentially expressed lincRNAs (DELs) were identified between tumor and normal tissues using the R package “Limma” with log2 | fold-change (FC) | > 1 and adjusted *P*-value < 0.05.

### The construction of lncRNAs-based prognostic signature

First, we used univariate and least absolute shrinkage and selection operator (LASSO) COX regression analysis to select the independent risk lncRNAs. Next, multivariate COX regression was used to identify corresponding coefficients of ESCC prognostic signature using R package “glment”, “survminer” and “survival”. The risk score of every patient from the TCGA and GEO cohorts were calculated based on the signature. All samples were randomly separated to high- and low-risk sets with the median score as cut-off value. Survival analysis for each set was evaluated by the Kaplan–Meier curve and log-rank test. The receiver operating characteristic (ROC) curve and the area under the curve (AUC) were drawn using R package “survivalROC”.

### Weighted gene co-expression network analysis (WGCNA)

WGCNA is a useful tool to establish the co-expression network between gene pattern and clinical factors using R package “WGCNA” (Version: 1.68). The procedure of WGCNA included identifying gene expression similarity matrix, adjacency matrix and co-expression network. ScaleFree plot was used for evaluating whether the network exhibits a scale free topology. The power value of soft threshold of adjacency matrix was identified as 5 to meet scale-free topology criterion. The hierarchically clustering analysis based on average-linkage were originated from the Dynamic using Tree Cut method for Branch Cutting (deep-split = 2, cut height = 0.4, minimum cluster size = 30). The association between modules and variables was identified to select relevant module.

### The nomogram establishing

A concise nomogram of predicting the survival of ESCC was established using the R package “rms”, “Hmisc”, “lattice”, “Formula”, and “foreign”. The concordance index (C-index) was performed to assess prediction capability. The patients with ESCC were separated to diverse risk clusters along with their scores. All analyses were completed with R software. The *P*-value < 0.05 was considered statistically significant.

### Data availability

All datasets generated for this study are included in the manuscript.

### Ethics statement

As the data (TCGA and GEO datasets) are publicly available, no ethical approval is required.

## References

[1] Bray F, Ferlay J, Soerjomataram I, Siegel RL, Torre LA, Jemal A. Global cancer statistics 2018: GLOBOCAN estimates of incidence and mortality worldwide for 36 cancers in 185 countries. CA Cancer J Clin. 2018; 68:394–424. 10.3322/caac.21492 30207593

[2] Abnet CC, Arnold M, Wei WQ. Epidemiology of Esophageal Squamous Cell Carcinoma. Gastroenterology. 2018; 154:360–73. 10.1053/j.gastro.2017.08.023 28823862 PMC5836473

[3] Arnold M, Soerjomataram I, Ferlay J, Forman D. Global incidence of oesophageal cancer by histological subtype in 2012. Gut. 2015; 64:381–87. 10.1136/gutjnl-2014-308124 25320104

[4] Rustgi AK, El-Serag HB. Esophageal carcinoma. N Engl J Med. 2014; 371:2499–509. 10.1056/NEJMra1314530 25539106

[5] Li QD, Li H, Wang MS, Diao TY, Zhou ZY, Fang QX, Yang FY, Li QH. Multi-susceptibility genes associated with the risk of the development stages of esophageal squamous cell cancer in Feicheng County. BMC Gastroenterol. 2011; 11:74. 10.1186/1471-230X-11-74 21672255 PMC3141752

[6] Schlemper RJ, Dawsey SM, Itabashi M, Iwashita A, Kato Y, Koike M, Lewin KJ, Riddell RH, Shimoda T, Sipponen P, Stolte M, Watanabe H. Differences in diagnostic criteria for esophageal squamous cell carcinoma between Japanese and Western pathologists. Cancer. 2000; 88:996–1006. 10.1002/(SICI)1097-0142(20000301)88:5<996::AID-CNCR8>3.0.CO;2-Q 10699887

[7] Shimizu M, Ban S, Odze RD. Squamous dysplasia and other precursor lesions related to esophageal squamous cell carcinoma. Gastroenterol Clin North Am. 2007; 36:797–811, v–vi. v-vi. 10.1016/j.gtc.2007.08.005 17996791

[8] Contino G, Vaughan TL, Whiteman D, Fitzgerald RC. The Evolving Genomic Landscape of Barrett’s Esophagus and Esophageal Adenocarcinoma. Gastroenterology. 2017; 153:657–673.e1. 10.1053/j.gastro.2017.07.007 28716721 PMC6025803

[9] Sugihara H, Ishimoto T, Miyake K, Izumi D, Baba Y, Yoshida N, Watanabe M, Baba H. Noncoding RNA Expression Aberration Is Associated with Cancer Progression and Is a Potential Biomarker in Esophageal Squamous Cell Carcinoma. Int J Mol Sci. 2015; 16:27824–34. 10.3390/ijms161126060 26610479 PMC4661918

[10] Li W, Zhang L, Guo B, Deng J, Wu S, Li F, Wang Y, Lu J, Zhou Y. Exosomal FMR1-AS1 facilitates maintaining cancer stem-like cell dynamic equilibrium via TLR7/NFκB/c-Myc signaling in female esophageal carcinoma. Mol Cancer. 2019; 18:22. 10.1186/s12943-019-0949-7 30736860 PMC6367809

[11] Xie JJ, Jiang YY, Jiang Y, Li CQ, Lim MC, An O, Mayakonda A, Ding LW, Long L, Sun C, Lin LH, Chen L, Wu JY, et al. Super-Enhancer-Driven Long Non-Coding RNA LINC01503, Regulated by TP63, Is Over-Expressed and Oncogenic in Squamous Cell Carcinoma. Gastroenterology. 2018; 154:2137–2151.e1. 10.1053/j.gastro.2018.02.018 29454790

[12] He J, Zhao H, Deng D, Wang Y, Zhang X, Zhao H, Xu Z. Screening of significant biomarkers related with prognosis of liver cancer by lncRNA-associated ceRNAs analysis. J Cell Physiol. 2020; 235:2464–77. 10.1002/jcp.29151 31502679

[13] Tang H, Wu Z, Zhang Y, Xia T, Liu D, Cai J, Ye Q. Identification and Function Analysis of a Five-Long Noncoding RNA Prognostic Signature for Endometrial Cancer Patients. DNA Cell Biol. 2019; 38:1480–98. 10.1089/dna.2019.4944 31539276

[14] Mou Y, Wang D, Xing R, Nie H, Mou Y, Zhang Y, Zhou X. Identification of long noncoding RNAs biomarkers in patients with hepatitis B virus-associated hepatocellular carcinoma. Cancer Biomark. 2018; 23:95–106. 10.3233/CBM-181424 29991128 PMC13078548

[15] Chen WJ, Tang RX, He RQ, Li DY, Liang L, Zeng JH, Hu XH, Ma J, Li SK, Chen G. Clinical roles of the aberrantly expressed lncRNAs in lung squamous cell carcinoma: a study based on RNA-sequencing and microarray data mining. Oncotarget. 2017; 8:61282–304. 10.18632/oncotarget.18058 28977863 PMC5617423

[16] Kouhsar M, Azimzadeh Jamalkandi S, Moeini A, Masoudi-Nejad A. Detection of novel biomarkers for early detection of Non-Muscle-Invasive Bladder Cancer using Competing Endogenous RNA network analysis. Sci Rep. 2019; 9:8434. 10.1038/s41598-019-44944-3 31182759 PMC6557814

[17] Xu TP, Huang MD, Xia R, Liu XX, Sun M, Yin L, Chen WM, Han L, Zhang EB, Kong R, De W, Shu YQ. Decreased expression of the long non-coding RNA FENDRR is associated with poor prognosis in gastric cancer and FENDRR regulates gastric cancer cell metastasis by affecting fibronectin1 expression. J Hematol Oncol. 2014; 7:63. 10.1186/s13045-014-0063-7 25167886 PMC4237812

[18] Liu Y, Xie D, He Z, Zheng L. Integrated analysis reveals five potential ceRNA biomarkers in human lung adenocarcinoma. PeerJ. 2019; 7:e6694. 10.7717/peerj.6694 31106044 PMC6497041

[19] Khalil AM, Guttman M, Huarte M, Garber M, Raj A, Rivea Morales D, Thomas K, Presser A, Bernstein BE, van Oudenaarden A, Regev A, Lander ES, Rinn JL. Many human large intergenic noncoding RNAs associate with chromatin-modifying complexes and affect gene expression. Proc Natl Acad Sci USA. 2009; 106:11667–72. 10.1073/pnas.0904715106 19571010 PMC2704857

[20] Zhang G, Wang Q, Zhang X, Ding Z, Liu R. LncRNA FENDRR suppresses the progression of NSCLC via regulating miR-761/TIMP2 axis. Biomed Pharmacother. 2019; 118:109309. 10.1016/j.biopha.2019.109309 31545237

[21] Huang GW, Xue YJ, Wu ZY, Xu XE, Wu JY, Cao HH, Zhu Y, He JZ, Li CQ, Li EM, Xu LY. A three-lncRNA signature predicts overall survival and disease-free survival in patients with esophageal squamous cell carcinoma. BMC Cancer. 2018; 18:147. 10.1186/s12885-018-4058-6 29409459 PMC5801805

[22] Mao Y, Fu Z, Zhang Y, Dong L, Zhang Y, Zhang Q, Li X, Liu J. A seven-lncRNA signature predicts overall survival in esophageal squamous cell carcinoma. Sci Rep. 2018; 8:8823. 10.1038/s41598-018-27307-2 29891973 PMC5995883

[23] Yu J, Wu X, Huang K, Zhu M, Zhang X, Zhang Y, Chen S, Xu X, Zhang Q. Bioinformatics identification of lncRNA biomarkers associated with the progression of esophageal squamous cell carcinoma. Mol Med Rep. 2019; 19:5309–20. 10.3892/mmr.2019.10213 31059058 PMC6522958

[24] Balachandran VP, Gonen M, Smith JJ, DeMatteo RP. Nomograms in oncology: more than meets the eye. Lancet Oncol. 2015; 16:e173–80. 10.1016/S1470-2045(14)71116-7 25846097 PMC4465353

[25] Wei JH, Feng ZH, Cao Y, Zhao HW, Chen ZH, Liao B, Wang Q, Han H, Zhang J, Xu YZ, Li B, Wu JT, Qu GM, et al. Predictive value of single-nucleotide polymorphism signature for recurrence in localised renal cell carcinoma: a retrospective analysis and multicentre validation study. Lancet Oncol. 2019; 20:591–600. 10.1016/S1470-2045(18)30932-X 30880070

[26] Lai J, Chen B, Zhang G, Wang Y, Mok H, Wen L, Pan Z, Su F, Liao N. Identification of a novel microRNA recurrence-related signature and risk stratification system in breast cancer. Aging (Albany NY). 2019; 11:7525–36. 10.18632/aging.102268 31548433 PMC6781975

[27] Zhou Z, Mo S, Dai W, Ying Z, Zhang L, Xiang W, Han L, Wang Z, Li Q, Wang R, Cai G. Development and Validation of an Autophagy Score Signature for the Prediction of Post-operative Survival in Colorectal Cancer. Front Oncol. 2019; 9:878. 10.3389/fonc.2019.00878 31552190 PMC6746211

[28] Liu Z, Li M, Hua Q, Li Y, Wang G. Identification of an eight-lncRNA prognostic model for breast cancer using WGCNA network analysis and a Cox-proportional hazards model based on L1-penalized estimation. Int J Mol Med. 2019; 44:1333–43. 10.3892/ijmm.2019.4303 31432096 PMC6713414

[29] Zhai X, Xue Q, Liu Q, Guo Y, Chen Z. Colon cancer recurrence-associated genes revealed by WGCNA co-expression network analysis. Mol Med Rep. 2017; 16:6499–505. 10.3892/mmr.2017.7412 28901407 PMC5865817

